# Effects of stance control *via* hidden Markov model-based gait phase detection on healthy users of an active hip-knee exoskeleton

**DOI:** 10.3389/fbioe.2023.1021525

**Published:** 2023-04-10

**Authors:** Miguel Sánchez-Manchola, Luis Arciniegas-Mayag, Marcela Múnera, Maxime Bourgain, Thomas Provot, Carlos A. Cifuentes

**Affiliations:** ^1^ Department of Biomedical Engineering, Colombian School of Engineering Julio Garavito, Bogotá, Colombia; ^2^ LabTel, Electrical Engineering Department at Federal University of Espírito Santo, Vitória, Brazil; ^3^ Bristol Robotics Laboratory, University of the West of England, Bristol, United Kingdom; ^4^ EPF Graduate School of Engineering, Cachan, France; ^5^ Arts et Métiers Institute of Technology, Institut de Biomécanique Humaine Georges Charpak, Paris, France; ^6^ School of Engineering, Science and Technology, Universidad Del Rosario, Bogotá, Colombia

**Keywords:** adaptive gait phase detection, assisted-as-needed, hidden markov model, lower-limb exoskeleton, robot-assisted gait training, stance control, stroke

## Abstract

**Introduction:** In the past years, robotic lower-limb exoskeletons have become a powerful tool to help clinicians improve the rehabilitation process of patients who have suffered from neurological disorders, such as stroke, by applying intensive and repetitive training. However, active subject participation is considered to be an important feature to promote neuroplasticity during gait training. To this end, the present study presents the performance assessment of the AGoRA exoskeleton, a stance-controlled wearable device designed to assist overground walking by unilaterally actuating the knee and hip joints.

**Methods:** The exoskeleton’s control approach relies on an admittance controller, that varies the system impedance according to the gait phase detected through an adaptive method based on a hidden Markov model. This strategy seeks to comply with the assistance-as-needed rationale, i.e., an assistive device should only intervene when the patient is in need by applying Human-Robot interaction (HRI). As a proof of concept of such a control strategy, a pilot study comparing three experimental conditions (i.e., unassisted, transparent mode, and stance control mode) was carried out to evaluate the exoskeleton’s short-term effects on the overground gait pattern of healthy subjects. Gait spatiotemporal parameters and lower-limb kinematics were captured using a 3D-motion analysis system Vicon during the walking trials.

**Results and Discussion:** By having found only significant differences between the actuated conditions and the unassisted condition in terms of gait velocity (*ρ* = 0.048) and knee flexion (*ρ* ≤ 0.001), the performance of the AGoRA exoskeleton seems to be comparable to those identified in previous studies found in the literature. This outcome also suggests that future efforts should focus on the improvement of the fastening system in pursuit of kinematic compatibility and enhanced compliance.

## 1 Introduction

Mobility is considered one of the most important human faculties which can be defined as the ability of an individual to move freely through multiple environments and perform activities of daily living with ease (Winter 2009). Following a neurological dysfunction, such as stroke, mobility may be affected and only a short-time period might remain to take advantage of the inherent adaptability and plasticity of the central nervous system for recovery (Wolpert et al., 2011). Reestablishing effective mobility for individuals with lower-limb impairments is often a complex challenge and frequently involves the interdisciplinary efforts of many medical, surgical, and rehabilitative specialists (Pasquina et al., 2017). Within this context, robot-based training has risen in the past years as a potential clinical aid for both patients and health professionals. Preliminary results on its influence on stroke survivors undergoing the sub-acute phase, i.e., within the first 3 months, suggest that combining rehabilitation devices with a conventional rehabilitation program appears to be more effective than overground gait training alone in the recovery of independent walking (Schwartz and Meiner, 2015; Mehrholz et al., 2017).

Most-commercially, available lower-limb assistive devices move the patient’s limbs along a predefined, fixed trajectory frequently drawn from healthy subjects (Knaepen et al., 2014). However, it has already been proven that a monotonous repetition of the same gait pattern results in decreased neuromuscular activity, increased energy consumption, and learned disuse (Taub et al., 2006; Knaepen et al., 2014). In order to induce motor learning based on the principle of neuroplasticity, studies have shown that therapy is only effective if task-oriented activities are performed whereby patient effort is promoted (Lagoda et al., 2012). Therefore, to interact with humans, wearable robots are expected to be flexible, adaptable, and, most importantly, safe. One of the benchmarks to achieve this is compliance. Compliance plays an important role in human adaptations to environmental changes and securing stable gait (Geyer et al., 2006). However, compliance has not yet found its way into commercial wearable robots, which usually use direct-drive actuation due to its high bandwidth and controllability (Bacek et al., 2018).

In pursuit of compliance, researchers have developed a new class of controllers to provide “assistance-as-needed” (AAN). For instance, strategies based on impedance modulation (Tsuji and Tanaka, 2005; Marchal-Crespo and Reinkensmeyer, 2009) help the subject only when away from a reference pattern (kinematic in most cases) by exerting proportional restoring forces. On the other hand, strategies based on proportional myoelectric control (Ferris et al., 2006) render control outputs that are directly proportional to the magnitude of surface electromyography (EMG) signals. However, this method relies on clean and reliable EMG acquisition from nominal, functional muscles which may be affected when working with patient groups (Dzeladini et al., 2016). Thus, the benchmark for the control of active lower-limb orthoses remains to be the impedance modulation. As a matter of fact, adaptive-impedance control strategies have proven to provide a gait training session as effective as that provided by physical therapists (Dzahir and Yamamoto, 2014).

Within this context, and based on the fact that humans change their joint impedance throughout gait by regulating their posture and muscle contraction levels for the sake of stability, some robotic devices use a particular strategy commonly known as stance control (SC). SC applies impedance modulation to provide stability and prevent the lower limb joints from collapsing during the stance phase, whereas it releases them to allow free movement during the swing phase (Ir and Azuan AO, 2015). Studies, which have used this strategy, have reported that it can increase walking speed, reduce energy expenditure and gait asymmetry (for both paretic and non-paretic limbs), thus decreasing muscle stress in patients with muscular weakness (Zissimopoulos et al., 2007; Zacharias and Kannenberg, 2012; Rafiaei et al., 2016).

In particular, Dzeladini et al. implemented an SC strategy based on neuromuscular control (NMC) to command an actuated ankle orthosis using few sensory inputs. Preliminary results involving two healthy subjects show a reduction in net metabolic cost and muscle activity, whereas walking dynamics remained relatively unchanged (Dzeladini et al., 2016). Conversely, similar to the current study, Villa-Parra et al. proposed a novel method to modulate the impedance on an active non-backdriveable knee orthosis by using variable gains which vary as a function of the user’s anthropometric measurements and gait phases extracted through footswitch signals. Both moment-based and velocity-based gain patterns appeared to be suitable to assist the knee joint under the SC strategy (Villa-Parra et al., 2017). Similarly, Ortlieb et al. featured a variable impedance controller that modulates its stiffness constant based on a 3-gait-phase model to actuate hip and knee joint along the sagittal plane during overground walking. Gait phases are detected through hip flexion velocity. Low compliance on a healthy user shows greater effects on the walking speed and step length (Ortlieb et al., 2019). Finally, the SC approach has also exhibited a positive influence on patients with paraplegia while used in hybrid neuroprostheses (HNP), i.e., the combination of functional neuromuscular stimulation (FNS) with a lower-limb orthosis. HNPs incorporating controllable knee mechanisms operating under the SC premise reduce the amount of stimulation required as compared to FNS systems only (To et al., 2011). Also, a pilot study involving one disabled subject shows that stance phase knee flexion is closer to the nominal condition (i.e., healthy walking) and knee hyperextension after initial contact decreases during walking with HNP compared to FNS-only gait (Bulea et al., 2013).

Other studies which vary the impedance in lower-limb joints focus on the rehabilitation or assistance of only one joint, particularly in the sagittal plane. For intance, by taking advantage of the fact that the swing phase requires free knee rotation, some quasi-passive devices have been developed and can be found in the literature (Shamaei et al., 2013a; Shamaei et al., 2014; Shamaei et al., 2014). Other studies feature devices that vary the knee stiffness by employing a spring system to generate loading profiles based on two gait phases detected by an instrumented insole. The implementation of compliant actuators (Cestari et al., 2015), described as a not-bulky device, and a two-phase gait detection method (namely, stance and swing phases) render three desirable stiffness states (i.e., Minimum, Medium, and High Stiffness). Further, a control strategy proposed for the HUALEX exoskeleton (Tran et al., 2016), featured a fuzzy-based impedance control strategy that changes the impedance applied by the exoskeleton to the user also for only two gait phases.

Even though the afore-mentioned studies highlight the promising results that the application of varying stiffness in active lower-limb devices have shown in the past years, most of the proposed control strategies consider only the main gait phases (i.e., stance and swing phases). Since the human hip and knee joint stiffness shows significant variations during the stance phase (Shamaei et al., 2013b), the control strategy output could be improved by using an online gait subphase detection with increased granularity. In addition, most studies apply robot-assisted gait training while assisting a single human joint, or rely on pressure sensors instrumented in customized insoles or multiple sensory inputs to divide the user’s gait cycle. Nonetheless, footswitch signals have demonstrated poor performance compared to, e.g., inertial sensors, on account of their short durability and the need for professional personnel to locate them precisely (Gouwanda and Gopalai, 2015). Also, although using multiple sensor interfaces seems to be reasonable for prosthetic devices (such as the one found in (Dzeladini et al., 2016)) since they are mounted to the prosthesis itself, in the case of orthoses, a minimal number of sensors should be pursued for the sake of clinical usability and the patient’s comfort (Gams et al., 2013). For these reasons, this work utilizes a more reliable, more versatile gait phase detection module (Sánchez Manchola et al., 2019) and a control strategy based on Stance Control (SC) to compensate for the rigid nature of the AGoRA exoskeleton. By combining these three main features: i) an adaptable inertial-based method to accurately segment the gait cycle, and ii) an impedance modulation performed according to the gait phase detected to adjust iii) a non-backdrivable hip-knee exoskeleton, the present study intends to find the short-term effects in terms of gait parameters and user perception of their interplay. Healthy subjects are involved in seeking a preliminary assessment of the proposed prototype as a means of debugging possible hard and software issues prior to trials with pathological individuals.

The document follows its track as shown hereby: Section 2 describes the hardware used in the active exoskeleton (together with an overview of its gait phase detection module and SC approach), followed by the experimental protocol, data processing, and statistical analysis of results. Subsequently, Section 3 presents the results in terms of gait spatio-temporal parameters and lower-limb kinematics, whereas Section 4 discusses the implications of such outcomes. Finally, Section 5 summarizes the findings and includes some recommendations to take into account in further iterations of the wearable device used.

## 2 Materials and methods

The proposed orthosis controller comprises two main components. The first (Section 2.2) is a Hidden Markov Model (HMM) that uses angular velocity components from a single Inertial Measurement Unit (IMU) placed on the user’s foot instep to accurately segment their gait cycle into four events of interest. The second component (Section 2.3.3) relies on an admittance controller, which works as a function of the detected gait phase, to generate a stance behavior on the active orthotic device whose hardware architecture is described as follows.

### 2.1 Active hip-knee exoskeleton

**FIGURE 1:**
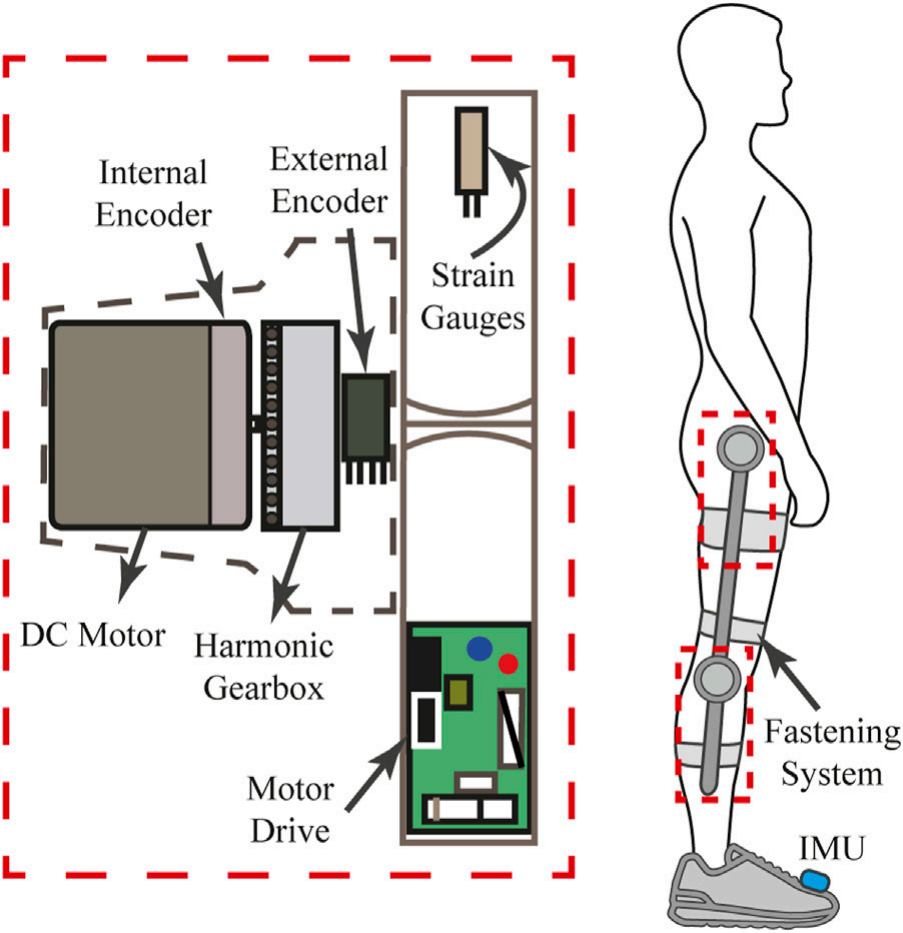
Schematic drawing of each joint assembly of the AGoRA exoskeleton.

The AGoRA exoskeleton is an active assistive device intended as a rehabilitation tool for stroke patients. It is mainly addressed for overground gait training in a clinical environment as a bilateral wearable device. The exoskeleton comprises two actuated Degrees of Freedom (DoF) to the hip and the knee flexion-extension (i.e., movement along the sagittal plane) as shown in Figure 1. An additional passive DoF in the hip joint allows the abduction/adduction movements for lateral balance support (Rietdyk et al., 1999). Also, most of its mechanical structure is made of duralumin: a light, high-resistant, and low corrosive material whose attributes are considered to be important in the design of wearable robots (Pons et al., 2008). These design criteria allow the device to have a total estimated weight of 15 *kg* with the actuation mechanisms mounted on both hip and knee joints. For further information on the mechanical structure of the AGoRA exoskeleton, you may refer (Sánchez-Manchola et al., 2018), where the entire infrastructure of the device is detailed.

Regarding the actuation mechanism used, a brushless flat DC motor (EC-60 flat 408057, Maxon Motor AG, Switzer land) is coupled with a harmonic drive gear (CSD-20-160-2AGR, Harmonic Drive LLC, United States) (see Figure 1) to actuate the hip and the knee joints of the AGoRA exoskeleton. This actuation system was chosen because this construction provides more torque at lower speeds (gear ratio of 160:1) while preserving lightweight and reduced volume (Zoss and Kazerooni, 2006). The proposed assembly provides a continuous net torque of 35 *Nm* and peak torques of 180 *Nm*, which meets the design requirements for most patients (Bayón et al., 2017). As control strategies meant to assist the human gait cycle do not require continuous torque but high torque profiles at specific times, the peak torque of the selected actuators appears to be sufficient for the application presented in this work (Colombo et al., 2000).

To command these actuation mechanisms, we opted for a distributed architecture with motor drives commanding each joint module independently for the sake of modularity. The exoskeleton joints were equipped with an EPOS4 driver (Maxon Motors AG, Switzerland) which has been developed by the manufacturer specifically for the brushless DC motors previously described (see Figure 1). The motor drives are in charge of the sensor data acquisition and provide three built-in low-level control loops: current, position, and velocity regulation. The data bus used to connect all functional modules consists of a network structure with a deterministic real-time communication based on Controller Area Network (CAN) technology running at 1 *Mbps*. (Bortole, 2014; Bayón et al., 2017). In order to power the overall architecture, a lithium-ion battery pack of 36 V_DC_ and 4.4 Ah is used while being kept inside a small backpack worn by the user.

Further, in pursuit of kinematic compatibility, i.e., the correct alignment of the exoskeleton hinges with the biological axes of rotation (Hughes et al., 2009), the length of each exoskeleton segment can be adjusted to different anthropometric measures without losing functionality. Via a mechanism of two telescopic bars, the thigh and shank segments can be adjusted to encompass a setting spectrum covering a target population whose height generally ranges from 1.70 to 1.83 *m* and whose maximum bodyweight may reach up to 90 *kg*. Additionally, adjustable rounded 3D-printed leg braces carriers with Velcro straps are used as a means of fastening (as may be seen in Figure 1).

In regards to sensors, the AGoRA exoskeleton is designed in such a way that there are no sensors physically attached to the subject’s skin. The exoskeleton is equipped with two types of sensors: kinematic and kinetic. Kinematic sensors are used for measuring hip and knee angular position, and foot angular velocity. Besides the internal relative incremental encoder of each DC motor (used for the implementation of position and impedance controllers), the exoskeleton has an absolute magnetic encoder placed concentrically to each joint assembly (see Figure 1). Conversely, kinetic sensors measure the interaction forces between the user’s limbs and the mechanical structure of the exoskeleton. In particular, strain gauges (632-180, RS Pro, United Kingdom) mounted on each exoskeleton link are used as force sensors (see Figure 1) while being bonded to its metal rods. These force sensors are connected in a half Wheatstone bridge configuration to enhance their measurement accuracy and to correct changes resulting from temperature variations. A commercial 24-bit Analog-Digital Converter for weighing scales (HX711, Avia Semiconductors, Czech Republic) is used to balance the bridge and to amplify the output 50 times. Its output signal covers torque measurements ranging from −40 to 40 *Nm*, thus complying with the maximum continuous torque of the actuators.

In addition to the above-mentioned sensors, an IMU (BNO055, BOSCH, Germany) placed on the dorsal side of the foot, which integrates a triaxial 14-bit accelerometer and an accurate close-loop triaxial 16-bit gyroscope, is used to accurately classify the gait cycle (see Figure 1). The IMU sensor is calibrated using a software library provided by the manufacturer which captures data while the following process is performed: the device is kept still on a flat surface for about 5 s (gyroscope calibration) and then it is rotated by 45-degree increments across one axis (accelerometer calibration). Despite conventional sensors (e.g., pressure sensors on customized insoles or motion capture systems) are widely used for experimental purposes, they are also well-known to be either too fragile for activities of daily living and difficult to set up, or limited to indoor applications (Smith et al., 2002; Attal et al., 2018). Thus, we decided to use inertial sensors over other sensors on the basis of their cost-effectiveness (Caldas et al., 2017) and the fact that inertial quantities present typical waveform features throughout a gait cycle (Taborri et al., 2016).

### 2.2 Gait phase detection

This detection module, presented in previous work (Sánchez Manchola et al., 2019), relies on the most widespread model for wearable robots, i.e., a four-event model, to segment the gait cycle into: i) the initial foot contact with the ground or Heel Strike (HS); ii) the loading response phase or Flat Foot (FF); iii) the heel lifting or Heel-Off (HO); and iv) the initial Swing Phase (SP) (Taborri et al., 2016). For the correct positioning of the inertial sensor, we opted for fastening it to the dorsal side of the foot because of the better performance that scalar classifiers have shown with the sensor placed in this location, even compared to other vectorial classifiers that involve further inertial signals captured from different lower-limb locations (Taborri et al., 2014). Thus, the foot instep poses a location that requires a minimum number of sensors and guarantees classification accuracy.

A Hidden Markov Model (HMM) is chosen to be the detection method for this particular application since the use of wearable sensors such as IMU sensors along with an algorithm based on HMM has shown high accuracy in the recognition of activities of daily living (Panahandeh et al., 2013; Bennett et al., 2016). HMM has even demonstrated to be more accurate in the context of motor activity recognition compared to different supervised and unsupervised methods such as k-means, Gaussian Mixture Model, Linear Discriminant Analysis, Dynamic Time Warping, and threshold-based algorithms (Mannini and Sabatini, 2010; Attal et al., 2015; Sánchez Manchola et al., 2019; Martin, 2020).

An HMM is a doubly stochastic process with *N* underlying discrete states that are not observable, i.e., its state sequence is hidden to the observer who only has access to the emissions of each state (Rabiner, 1989). The second embedded stochastic process describes the emissions from *Y* observations, i.e., either the sensor readout or feature vectors extracted from them, in terms of discrete probabilities or probability density functions (Rabiner, 1989). HMM is a statistical model widely used to estimate a sequence of hidden states in a time series (Taborri et al., 2015), which for the case of gait phase detection corresponds to the gait events (*N* = 4, for this case).

HMM can be expressed as a function of a set *λ* of statistical measures:

$$\lambda =\left(A,B,\pi \right)$$ (1)

which includes the probability distribution matrix of state transition *A*, the probability distribution matrix of observation symbols *B*, and the initial state distribution vector *π*.

The development of a continuous HMM entails two main procedures: a training stage and a test stage. The first phase refers to the adjustment of model parameters *λ* to optimally adapt them to an observed training dataset (Rabiner, 1989). The Baum-Welch algorithm, the most common solution to this issue, is implemented in the present work to optimize the transition and emission probabilities starting from an initial parameter set (Sánchez Manchola et al., 2019). Subsequently, the test stage allows the feature classification based on the trained model achieved in the training phase, i.e., the search for the optimal state sequence is undertaken. The Viterbi algorithm represents a widely used optimality criterion to tackle this testing procedure (Rabiner, 1989). However, and despite its computational efficiency, this algorithm is not suitable for real-time implementation since the indicators it uses are computed based on a whole observation dataset (Sánchez Manchola et al., 2019). Thus, its real-time implementation becomes fundamental for it to be used in a lower-limb rehabilitation robot.

On this basis, a new online decoding approach known as the Forward-Only Viterbi (FOV) has been implemented to overcome this limitation. The FOV algorithm is applied to each signal in order to find the *l*-th state of likely sequence at a certain time *t*_*n*_ $\left(l_{t_{n}}\right)$ and the probability associated at each *i*-th state $\delta_{t_{n}}\left(i\right)$ (Taborri et al., 2014). Particularly, this decoding approach can be deployed by implementing the pseudo-code presented in Algorithm 1. A study performed by Mannini and Sabatini, however, found that this Viterbi decoding alternative is plagued by erroneous events that consist of missed and additionally detected gait strides Mannini and Sabatini (2012). Contrary to the mentioned study, in which a heuristic strategy discarded detected gait strides if their time duration was less than 250 ms, we do not take into account gait phases that last less than 150 ms (i.e., the shortest period for HS in healthy subjects during comfortable walking (Lemke et al., 2000)).

Algorithm 1 FOV algorithm

Require:
1: procedureInitialization
2: $\delta_{t_{0}}\left(i\right)\leftarrow \pi_{i}b_{i}\left(Y\left(t_{0}\right)\right),\;1\leq i\leq N$ ;
3: $l_{t_{0}}\leftarrow {\mathrm{argmax}}_{1\leq i\leq N}\delta_{t_{0}}\left(i\right)$ ;
4: end procedure
Ensure: $l_{t_{n}}$

5: for each new frame *b* do
6: $\delta_{t_{n}}\left(i\right)\leftarrow \max\left(\delta_{t_{n-1}}\left(i\right)A_{ij}\right)\times B_{i}\left(Y\left(t_{n}\right)\right)$ ;
7: $l_{t_{n}}\leftarrow {\mathrm{argmax}}_{1\leq i\leq N}\delta_{t_{n}}\left(i\right)$ ;
8: end for

The HMM algorithm applied in this study (Sánchez Manchola et al., 2019) was assessed using a custom insole as a reference system. The custom insole comprised four force-sensitive resistors placed in the hallux, the first and fifth metatarsophalangeal, and the heel. These pressure centers are selected because they show unique pattern characteristics in the estimation of ground reaction forces (Lim et al., 2017). After using standardized parameters training (SPT), the system showed an accuracy of 81.4% in healthy users and 78.06% in patients who had suffered some pathology that affected their gait pattern. A complete description of this gait phase detection module and its validation is available in (Sánchez Manchola et al., 2019).

### 2.3 Control architecture

**FIGURE 2:**
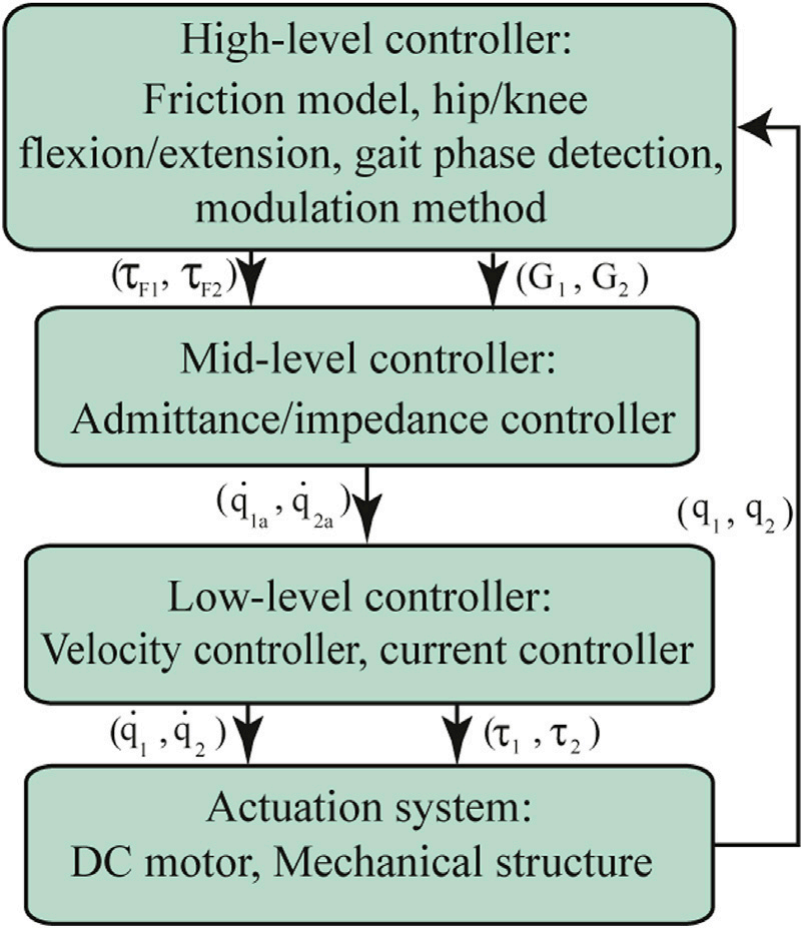
The control architecture of the AGoRA exoskeleton is comprised of 3 control levels: a high-level controller, comprised by a friction model with a resulting compensation torque *τ*_*F*_ and a modulation method that calculates the admittance controller gains according to each gait phase detected (*G*); a mid-level controller which uses an admittance/impedance controller, where ${\dot{q}}_{n}$ is the resulting angular velocity profile; and a low-level controller that involves a speed controller and a current controller, where $\dot{q}$ is the resulting angular velocity and *τ* corresponds to the low-level controller output. Subindexes 1 and 2 correspond to the hip and knee joints, respectively.

Human-Robot Interaction (HRI) involves different control architectures to assist a user’s lower limbs. In recent decades, multiple lower limb exoskeletons have been designed with a multi-layer control architecture (Minchala et al., 2017; Al-Shuka et al., 2019; Kumar et al., 2019; Lee et al., 2020). This control architecture is usually composed of a low-level controller comparable to standard controllers such as PID, PI, and PD. This controller changes based on the desired activity that the user intends to execute (Tijjani et al., 2022). The mid-level controller is used for two tasks: in the first one, a standard controller applies the control strategy; and in the second one, mathematical equations are considered to estimate the HRI and convert it into supported values used by the low-level controller (Vantilt et al., 2019). Finally, a high-level controller may involve machine-learning methods to calculate the control gain variations in accordance with the user’s desired activity (Minchala et al., 2017; Vantilt et al., 2019). For the case of the AGoRA exoskeleton, its control architecture comprises a multilevel control structure which encompasses the gait pattern estimation, the torque/force or angular values estimation, and the standard controllers to apply estimated parameters on the exoskeleton actuation system (Figure 2). This section shows the AGoRA exoskeleton’s multilevel control architecture for the SC application.

#### 2.3.1 Low-level controller

This module comprises a PI speed controller and a PI current controller. The velocity control has been used as part of the admittance controller to express the user’s motion intention in terms of angular velocity values. The outcome of the admittance controller is thus used as an input for the speed controller which commands the exoskeleton’s actuation mechanism.

**FIGURE 3:**
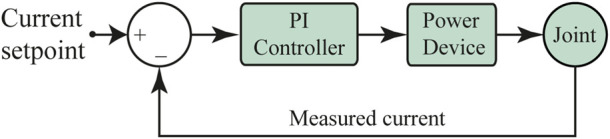
Schematic of the PI current controller implemented in the AGoRA exoskeleton as a low-level controller.

**FIGURE 4:**
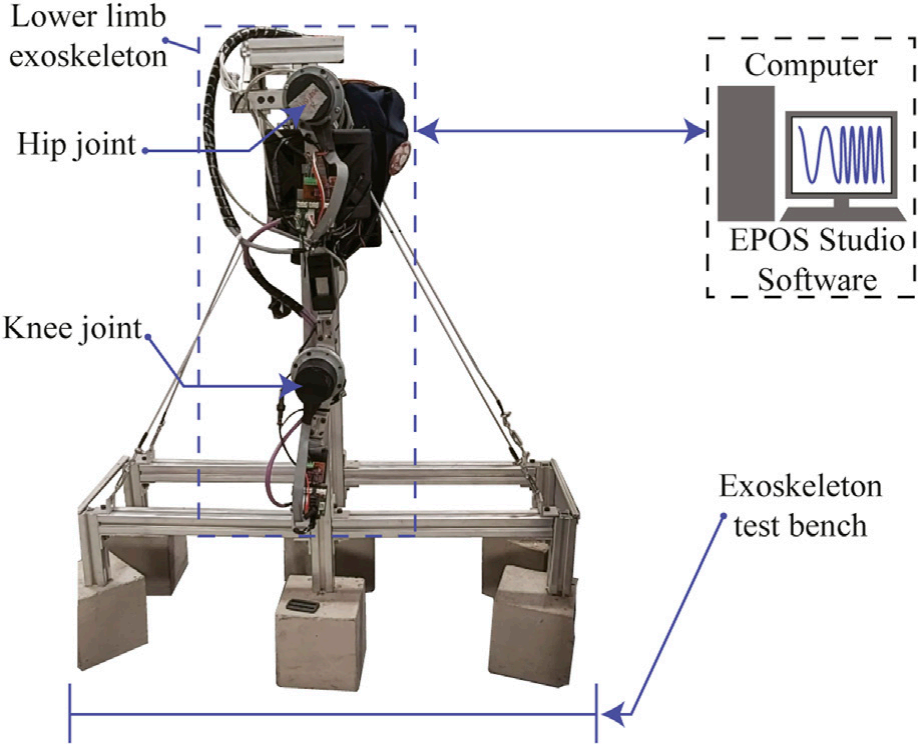
Experimental setup for the gain tuning of the AGoRA exoskeleton’s low-level controller.

The velocity controller internally uses a PI current controller which comes as an out-of-the-box tool on the Maxon’s EPOS4 driver. Figure 3 shows a simplified model of this current controller. Its corresponding gains were estimated by using the EPOS Studio software (Maxon Motors, v.3.2) which provides an auto-tuning option, where a chirp signal is applied to the actuation mechanism. For this tuning process, the exoskeleton was coupled to a rigid structure (test bench) to allow free hip and knee movements, as shown in Figure 4. As a result, the following PI were obtained: *p* = 955671 *μV*/*A*, *I* = 485188 *μV*/*As* for the hip joint, and *p* = 937294 *μV*/*A*, *I* = 430053 *μV*/*As* for the knee joint.

**FIGURE 5:**
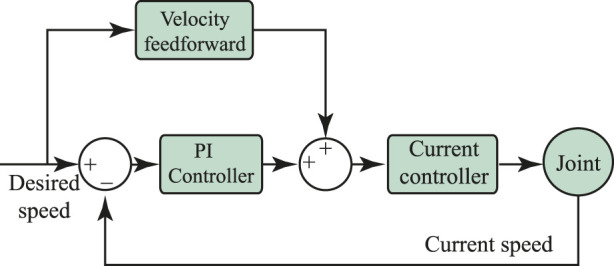
Schematic of the PI speed controller implemented in the AGoRA exoskeleton as a low-level controller.

A velocity controller is also a built-in tool of the EPOS4 driver and its schematic is shown in Figure 5. The characterization of the corresponding PI gains was performed in a similar way as for the current controller and the resulting values are *p* = 385000 *muAs*/*rad*, *I* = 4515922 *muA*/*rad* for the hip joint, and *p* = 427769 *μAs*/*rad*, *I* = 4442130 *μA*/*rad* for the knee joint.

#### 2.3.2 Mid-level controller

Even though the actuation mechanism used for the AGoRA exoskeleton can exert torque profiles which are sufficient to assist human lower limbs (as depicted in Section 2.1), the motor-gearbox assembly employed in each exoskeleton joint does not allow the unpowered hardware to comply with the user’s command. Since such behavior could eventually cause injuries to the patient by restricting their natural gait pattern, the implementation of compliant control strategies appears to be necessary to mitigate these adverse effects.

The admittance controllers have proven to be stable in high stiffness conditions (del Ama Espinosa, 2013). An admittance controller is a variation of the impedance controller (designed by Hogan in 1984 (Hogan, 1984)) whose performance highly depends on the actuator position and the actuator velocity. If the mechanical impedance of the exoskeleton could be zero (infinite admittance), its user would not feel any resistance while wearing it. However, this zero-impedance behavior is only ideal, given the actuator’s intrinsic inertia and friction, and the controller time delay (Hoogen et al., 2002). Low impedance, nonetheless, can be achieved if the control system takes into account the user’s motion intention. This low impedance behavior is widely known as backdrivability (Krebs et al., 2000), and good backdrivability provides numerous benefits in robot-assisted gait training, e.g., the ability to act as a monitoring tool for health professionals (Ichinose et al., 2003). In particular, an admittance controller is implemented in the AGoRA exoskeleton by modeling both the hip and the knee joints as a mass-damper system. In this sense, the motion of each exoskeleton joint will depend on its angular velocity and a damping constant, resulting in a system that simulates stiffness as shown in Eq. (2). Here, *D* is the damping constant, *v* is the angular velocity differential, and *F*_*d*_ is the force generated by the virtual damping.

$$Dv=F_{d}$$ (2)

**FIGURE 6:**
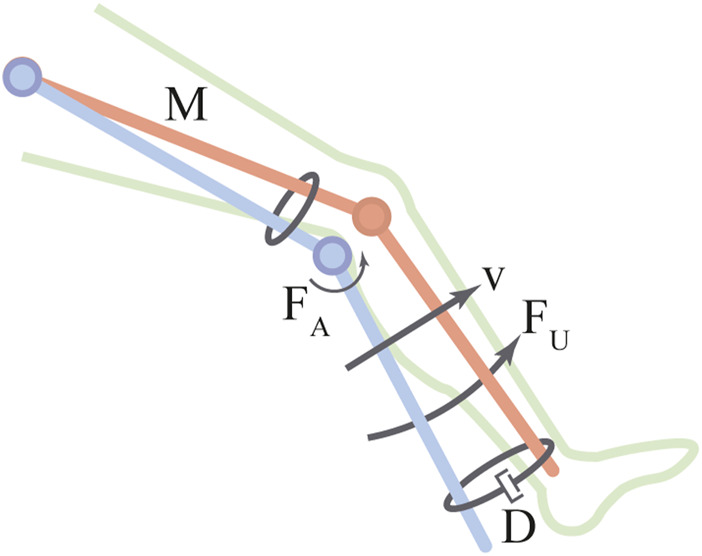
Schematic of mass-damper system.

The dynamic behavior of the HRI present in the AGoRA exoskeleton can be modeled as shown in Figure 6. In this model, the exoskeleton is assumed to have a given mass *M* and a damping constant *D*. Thus, the equation that describes the system velocity (*v*) is given by Eq. (3).

$$M\dot{v}+Dv=F_{i}$$ (3)

The interaction force *F*_*i*_ is measured by the strain gauges used as force sensors. In the frequency domain, Eq. (3) can be expressed as in Eq. (4).

$$v\left(s\right)=F_{i}/\left(Ms+D\right)$$ (4)

The output velocity value is passed through the low-level velocity controller (embedded within the motor drive) as an input. Using Eq. (4), the rendered admittance (*Y*) can be modeled as in Eq. (5).

$$Y\left(s\right)={\left(Ms+D\right)}^{-1}$$ (5)

**FIGURE 7:**
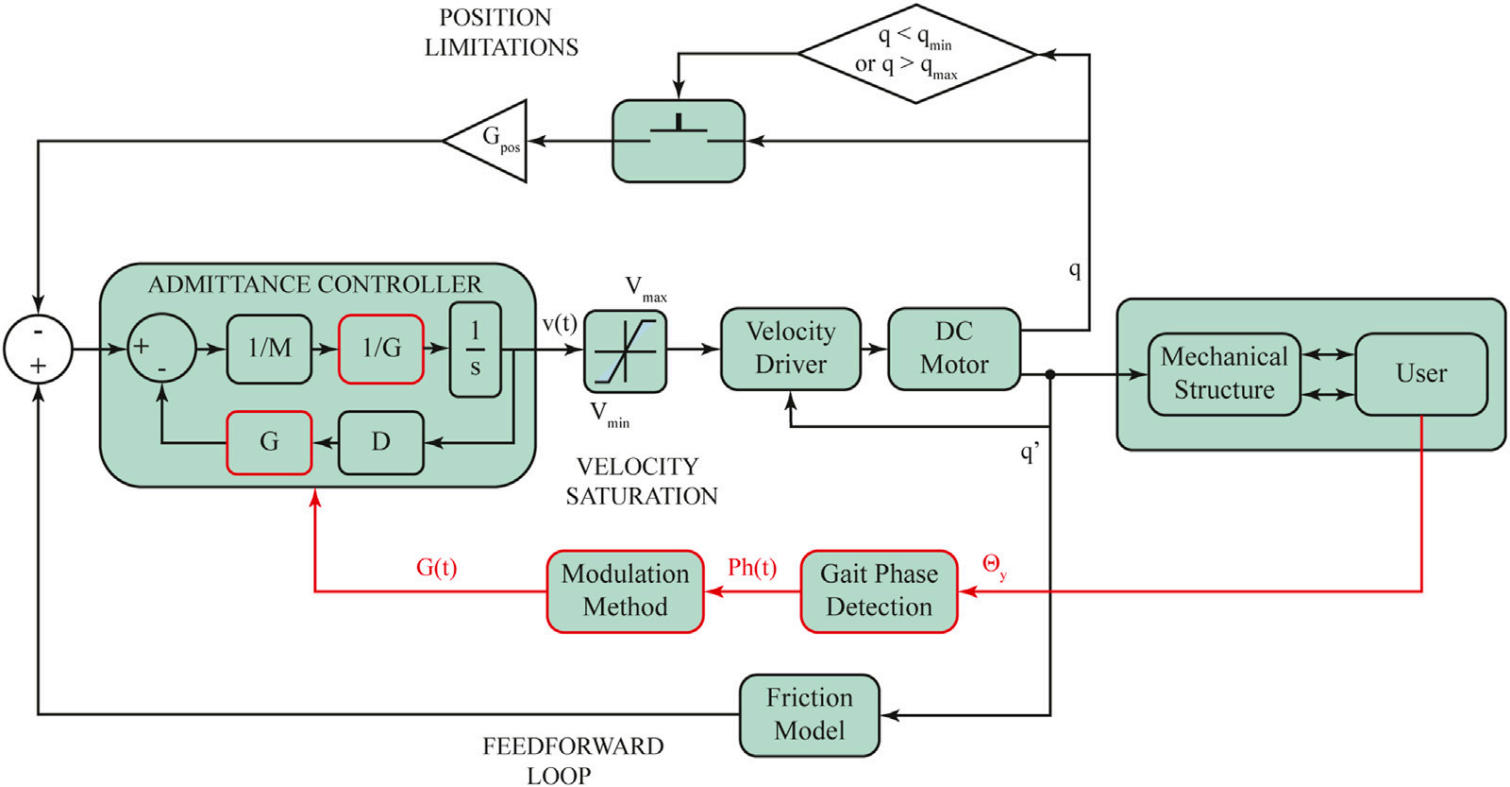
Stance control strategy implemented in the AGoRA exoskeleton. This control approach takes into account the human-robot interaction forces to render limited velocity profiles accordingly. Additional position limitations are included for the sake of safety. Θ_*y*_: Angular velocity along the sagittal plane. *Ph*(*t*): Current detected gait phase as a function of time.

The rationale behind the admittance control is thus to make the actuation mechanism show low impedance (high admittance) when moved by the patient’s extremities (see Admittance Controller in Figure 7). The admittance controller receives the calculated forces mentioned in Eq. (4) as input and renders velocity values accordingly as output. The impedance parameters *M* and *D* are set to 0.25 *kg* and 2.5 *N*/(*m*/*s*), respectively. These values were obtained empirically during tests undertaken on a test bench and adjusted for each exoskeleton joint during preliminary walking trials.

#### 2.3.3 High-level controller

A feedforward control loop is included within the control scheme to anticipate and compensate the dynamics of the exoskeleton structure. By modeling the exoskeleton as a 2-DoF robot, a friction model may be proposed (see *Feedforward Loop* in Figure 7). The friction model (Wu et al., 2016) depicts the torque value the actuation mechanism needs to exert to compensate for the influence of friction. Eq. (6) defines the proposed friction model, where *F*_*c*_ is the Coulomb friction, $\dot{q}$ is the joint’s angular velocity, and *T*_*m*_ and *F*_*s*_ are the starting torque and the static friction torque, respectively, which are defined using the gearbox datasheet.

$$T_{f}=\left\{\begin{array}{c} F_{c}\;\text{sgn}\left(\dot{q}\right)\;\dot{q}\neq 0\\ T_{m},|T_{m}|<|F_{s}|\;\dot{q}\approx 0\\ \left.F_{s},|T_{m}|\geq F_{s}\right)\;\dot{q}\approx 0 \end{array}\right.$$ (6)

Further, to avoid exceeding joint limits and subsequently causing damage or instability to the user, limitations in terms of position (*q*_*min*_, *q*_*max*_) and velocity (*V*_*min*_, *V*_*max*_) are also taken into consideration (as depicted as *Position Limitations* and *Velocity Saturation* respectively, in Figure 7).

**FIGURE 8:**
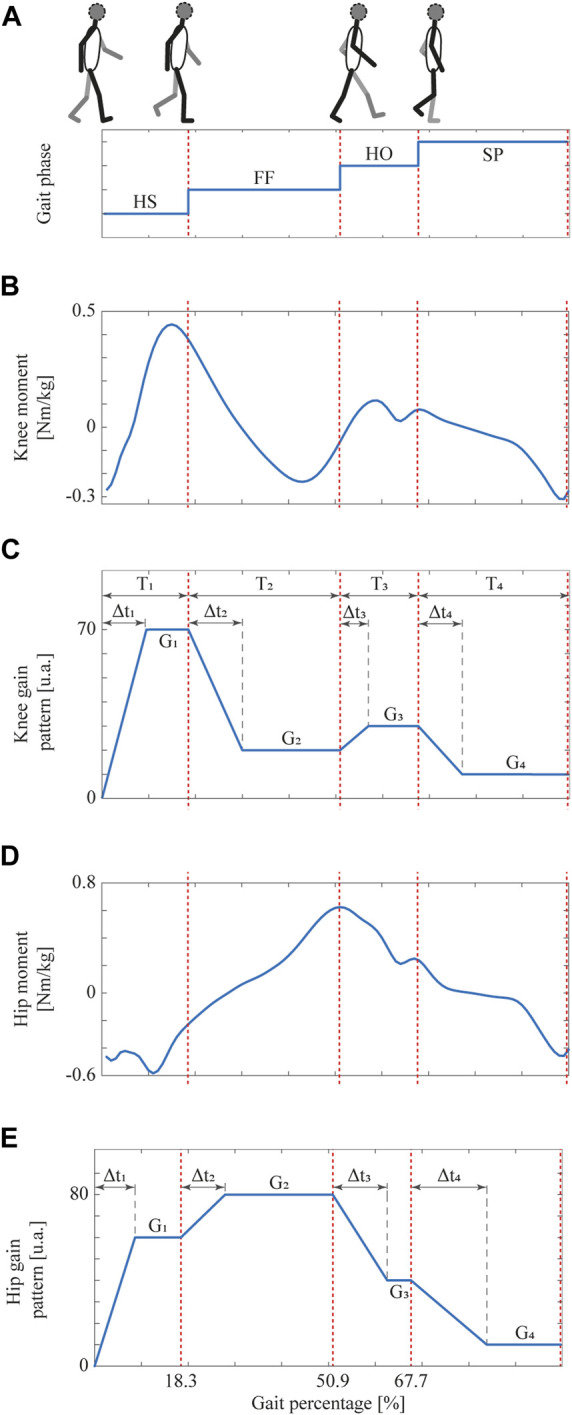
The gait phase detected schematic during the walking activity, taking into account the time spans (Δ*t*_*i*_) for each gait phase.; **(A)** Gait phases detected by means of the inertial-based partitioning method. **(B)** Knee and **(D)** Hip moment throughout the gait cycle in healthy subjects at a comfortable speed (Fukuchi et al., 2018). Also, gain patterns based on the **(C)** knee and **(E)** hip moment which ensures smooth transitions between gait phases. Adapted from (Villa-Parra et al., 2017).

In order to deploy an SC strategy with the admittance controller already described, an impedance modulation is achieved by directly multiplying a variable gain *G* by the controller constants *M* and *D* (as shown in red in Figure 7) (Villa-Parra et al., 2017). *G* is directly proportional to the system impedance and is updated according to the different gait sub-phases to adapt both joint impedances during gait. When varying the controller gain *G* in terms of four gait partitions (as shown in Figure 8A), typical lower limb moment during gait (as shown in Figures 8B, D, for knee and hip joints, respectively) should be displayed. In this context, *G* is defined and smoothly varied for each detected gait phase *i* following a pattern based on typical moment variations throughout gait (as reported in a public dataset of overground walking kinematics and kinetics in healthy subjects (Fukuchi et al., 2018)). Figures 8C, E show an example of such *G* variations within a single gait cycle for the knee and hip joints, respectively. And so, for the case of the knee joint, the highest gain value is given during HS when the knee experiences its first flexion, and then it gradually decreases with a slight increase during HO (as found by (Villa-Parra et al., 2017)). Following the same premise applied to the knee joint, the impedance modulation for the hip joint keeps the controller gain at high values throughout the stance phase, while there is a significant gain decrease during SP, in pursuit of stability during loading response. For both patterns, the proposed impedance modulation seeks to promote a shock-damping behavior during gait phases associated with weight acceptance (i.e., HS and FF where knee and hip apply a large moment value).

Regardless of the exoskeleton joint, *G*_*i*_, 1 ≤ *i* ≤ 4 require suitable time spans Δ*t*_*i*_, 1 ≤ *i* ≤ 4, in which the controller gain linearly decreases/increases until it reaches the desired value for HS, FF, HO, and SP, respectively. Such linear change allows the admittance controller to exert smooth velocity profiles according to each subject’s gait pattern. Considering that the weight and gait velocity are the anthropometric measures that most affect the knee mechanical behavior (Shamaei and Dollar, 2011), these parameters are considered here to define the corresponding *G* and Δ*t* values (Villa-Parra et al., 2017).

More specifically, time spans Δ*t*_*i*_ are established using Eq. (7) which defines these time periods as a function of known physical properties: the corresponding gait phase (*i*), the sampling frequency (*f*_*s*_), and the user’s height (*H*) and velocity (*v*_*u*_) (Villa-Parra et al., 2017).

$$\Delta t_{i}=\frac{0.0413\cdot i\cdot H\cdot f_{s}}{v_{u}}$$ (7)

Moreover, knee moment-based pattern (shown in Figure 8C) is configured to reach the following gain values: *G*_1_ = 0.7 ⋅ *W*, *G*_2_ = 0.2 ⋅ *W*, *G*_3_ = 0.3 ⋅ *W* and *G*_4_ = 0.1 ⋅ *W*, whereas hip moment-based pattern (shown in Figure 8E) is set to follow these gain values: *G*_1_ = 0.6 ⋅ *W*, *G*_2_ = 0.8 ⋅ *W*, *G*_3_ = 0.4 ⋅ *W* and *G*_4_ = 0.1 ⋅ *W*, with *W* being the user’s weight in kg.

Algorithm 2 shows the pseudo-code implemented in the Robot Operative System (ROS) for the online gain pattern generation (see *Modulation Method* in Figure 7), where *Ph*_*d*_ is the default phase from which the pattern begins to be generated, *Ph*_*c*_ is the current gait phase detected through the inertial-based algorithm, and Δ*G* is the gain increment added every sample.

Algorithm 2 Online Gait Pattern Modulation

Require:
1: procedure Initialization
2: *G*_*i*_; Δ*t*_*i*_ ← (0.0413 ⋅ *i* ⋅ *H* ⋅ *f*_*s*_)/*v*_*u*_;
3: $Ph\leftarrow Ph_{d};\;G\leftarrow G_{Ph_{d}}$ ;
4: *step* ← 1; Δ*G* ← 0;
5: end procedure
Ensure: *G*
6: for each new phase *Ph* do
7: if *Ph*_*c*_ ≠ *Ph* then
8: $\Delta G\leftarrow \left(G_{Ph_{c}}-G\right)/\Delta t_{Ph_{c}}$ ;
9: *step* ← 1;
10: *Ph* ← *Ph*_*c*_;
11: else
12: *G* ← *G* + Δ*G*;
13: *step* ← *step* + 1;
14: if *step* > Δ*t*_*Ph*_ then
15: Δ*G* ← 0;
16: end if
17: end if
18: end for

### 2.4 Experimental protocol

The experimental protocol comprises two phases to evaluate the SC response during the walking activity. First, a preliminary test was carried out on a treadmill to assess the SC response in terms of angular hip position, angular knee position, detected gait phase, and the calculated SC gains for the knee and the hip joints. Second, an overground walking test was implemented to assess the gait spatiotemporal parameters in healthy subjects using the SC in the AGoRA exoskeleton. The details about each test are featured bellow.

#### 2.4.1 SC test in treadmill

The stance controller was tested through a pilot study that involved a healthy user (i.e., they did not suffer from any gait-associated pathology) who used the AGoRA exoskeleton while walking over a conventional treadmill. A six-minutes walking test (6MWT) was performed on the treadmill at $\frac{3Km}{h}$ (such a speed was determined during a preliminary 10-m walking test applied to the user). During this preliminary trial, the angular position, angular velocity, detected gait phase, and computed controller gains were obtained for both hip and knee joints. Data acquisition was executed through sensor recording features available within ROS.

#### 2.4.2 SC test during overground walking

**FIGURE 9:**
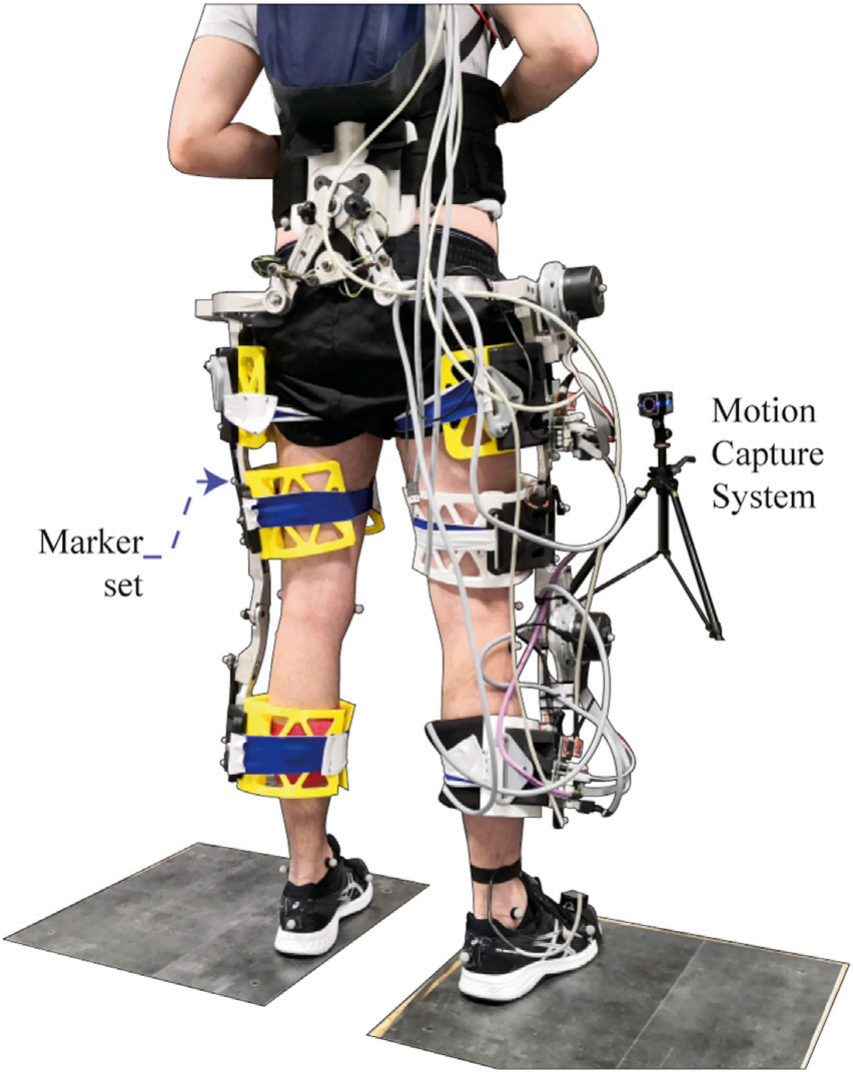
Experimental setup for trials with healthy subjects wearing the AGoRA exoskeleton.

A pilot study was conducted involving six neurologically intact subjects (6 males, 25.5 ± 6.1 y.o., height of 1.8 ± 0.03 *m*, and weight of 71.5 ± 10.9 *kg*) to assess the SC influence on spatiotemporal parameters and lower-limbs kinematics in healthy subjects during exoskeleton-assisted overground gait. A unilateral version of the exoskeleton was used for this study, i.e., the right hip and knee joints were actuated while the left leg was only attached to the mechanical frame (no actuation mechanisms were assembled), as may be seen in Figure 9.

At the beginning of the test, the subject’s anthropometric features were measured to adjust the exoskeleton segments accordingly and to set up the initial parameters of the admittance controller on the basis of the user’s weight and height. The user then performed a 10-m overground walking test while wearing the IMU on the tip of their dominant foot and the acquisition module on their back for the training stage of the gait phase detection module. Subsequently, the AGoRA exoskeleton was mounted on the volunteer by attaching the Velcro straps to their limbs and donning the backpack containing the battery and the main board. An emergency button was then handed to the subject, as he was instructed to press it in any situation that might compromise their safety or comfort to completely shut down the device. Once the exoskeleton was already worn and powered, the user was encouraged to walk for a short period (not longer than 10 min) so he would accommodate the device. Ten level-ground 10-m walking trials were subsequently performed for each of the three experimental conditions: i) a Transparent Mode (TM) that used the admittance control described in Section 2.3.2 to compensate for the rigid nature of the unpowered device, ii) the actual SC mode, and iii) the unassisted mode (baseline level of this study) assessed after the exoskeleton was removed from the volunteer. The two initial conditions were randomized for each subject in such a way that they were not aware of which operation mode they were experiencing. All subjects were encouraged to walk at their self-selected comfortable speed, while different kinematic and kinetic data were captured. A 3D-motion analysis system Vicon, equipped with 12 high-speed infrared cameras (Vicon Motion Systems Ltd., Vicon-Oxford, United Kingdom), was used to monitor human and exoskeleton joint angular displacements at a sampling rate of 200 Hz. The marker set consisted of 27 reflective markers (configuration adapted from (Bourgain et al., 2018) and focused on lower limbs and pelvis), thus allowing a lower-limb analysis in an indoor analysis laboratory (see Figure 9). The subject was instructed to tap the floor once firmly at the beginning of each trial so that a distinctive peak was recognizable by both measurement systems as a means of data synchronization.

The experimental protocol, including all the proposed procedures involving healthy subjects, were approved by the local Ethics Committee at the Colombian School of Engineering. All participants recruited for this study signed a written informed consent in which they stated to be aware of the possible risks they were facing while undergoing these trials and agreed to participate in spite of them. The volunteers of this study were selected based on their health status and physical conditions by taking into account the inclusion criteria listed below.

• Male able-bodied adults aged between 18 and 65 y.o.
• No history of a neurological, neuromuscular or physical disability that may hinder their normal gait pattern.
• Height within the range of 170–185 cm.
• Weight not greater than 90 kg.
• Some specific anthropometric measurements:• Femur length: 42–48 cm.• Distance between trochanters: 32–37 cm.• Tibia length: 28–31 cm.

These criteria were established based on the functional range within which the exoskeleton can be adjusted (see Section 2.1).

### 2.5 Data processing

Marker trajectories were first smoothed with an average sliding window (five values) with two passes in reverse direction to minimize the shifting effect. Any gaps in the raw motion data were filled using a C2-spline interpolation (gaps shorter than 15 frames) within the Vicon’s software *Nexus* (Hybois et al., 2019). Marker trajectories and kinetic data were then imported into OpenSim v.3.3. software and processed through a multibody kinematic optimization technique. To this end, a lower-limb model was implemented based on Raabe’s model (Raabe and Chaudhari, 2016) to generate a generic model with seven segments and 12 generalized coordinates. The generic model was then scaled to each participant on the basis of a static acquisition captured before all walking trials. All trials were finally processed using the final model and the inverse kinematics tool available on the OpenSim software in order to obtain gait spatiotemporal parameters and hip-knee kinematics (Zacharias and Kannenberg, 2012; Knaepen et al., 2014).

### 2.6 Statistical and user’s satisfaction analysis

Regarding statistical analysis, gait spatiotemporal parameters (e.g., step length, cadence, *etc.*) and hip and knee flexion/extension values from the six involved subjects were loaded into the SPSS software v.23.0 (IBM-SPSS Inc., Armonk, NY, United States), and either one-way repeated-measures ANOVA tests were conducted to compare among experimental conditions with a statistical significance level of *ρ* < 0.05 or Friedman tests (non-parametric version of ANOVA test) depending on whether the data exhibited a normal distribution or not (Shapiro-Wilk test). Bonferroni’s tests were carried out as a *post hoc* test in case significant differences were found.

Finally, a questionnaire (adapted Quebec User Evaluation of Satisfaction with Assistive Technology, QUEST 2.0) was used to evaluate the subject’s perception towards the assistive device (Demers et al., 2002). Only the questions related to assistive technology (e.g., weight, safety, durability, simplicity of use, and comfort) were used in this study since the AGoRA exoskeleton is still undergoing validation stages. The score for each question ranges from 1 to 5 (1: not satisfied at all; 2: not very satisfied; 3: more or less satisfied; 4: quite satisfied; and 5: very satisfied), and a final score is obtained as the median ± the interquartile range of the valid responses.

## 3 Results

### 3.1 SC response during treadmill walking

**FIGURE 10:**
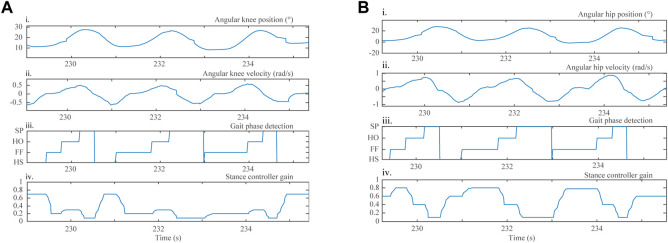
Three gait cycles of a healthy subject during a 6-min treadmill walking test, in terms of the following parameters: (i) angular joint position; (ii) angular joint velocity acquired with the AGoRA exoskeleton incremental encoders; (iii) gait phase detection outcome, determined using the gait phase detection module; and (iv) modulation method response, for the **(A)** knee and **(B)** hip joints.

Figure 10 shows the system response under treadmill walking conditions in terms of angular position, angular velocity, the gait phase detected through the inertial-based method, and the resulting controller gain across few gait cycles for both hip and knee exoskeleton joints. Such outcome exhibits angular position (Figure 10Ai; Figure 10Bi) and angular velocity values (Figure 10Aii; Figure 10Bii) which are typical for healthy subjects (Knaepen et al., 2014) and the way the modulation method (described Section 2.3.3 and shown in Figure 10Aiii; Figure 10Biii) successfully renders reasonable gain values for the admittance controller (described in Section 2.3.3 and shown in Figure 10Aiv; Figure 10Biv).

### 3.2 Gait parameters during overground walking

Spatiotemporal gait parameters among the three experimental conditions, i.e., unassisted, TM, and SC, are presented in Table 1. For the six involved participants, these results only demonstrate a significant difference from the unassisted condition for the case of gait velocity while walking in SC (*ρ* = 0.048). Conversely, the other spatiotemporal parameters show no significant difference from the unconstrained baseline (i.e., unassisted condition) which lies close to typical gait parameters found previously for healthy subjects (Knaepen et al., 2014).

**TABLE 1:** Spatiotemporal gait parameters calculated by the 3D-motion analysis system Vicon for all experimental conditions assessed: Unassisted, Transparent Mode (TM), and Stance Control (SC) (mean ± std). Asterisks indicate significant differences from the unassisted condition (Bonferroni, *p* <0.05).

Parameter	Unassisted	TM	SC
Gait velocity [m/s]	1.12 ± 0.34	0.78 ± 0.35	0.66 ± 0.23*
Cadence [steps/min]	83.38 ± 21.71	61.95 ± 21.21	58.77 ± 18.33
Stride length [cm]	161.68 ± 9.47	146.88 ± 20.91	135.88 ± 27.77
Step duration (right leg) [s]	0.90 ± 0.46	1.16 ± 0.60	1.15 ± 0.65
Step duration (left leg) [s]	0.70 ± 0.096	1.06 ± 0.51	1.14 ± 0.4
Stride duration [s]	1.59 ± 0.51	2.21 ± 1.02	2.28 ± 0.94
Stance phase (right leg) [%]	53.8 ± 9.52	50.32 ± 11.9	48.28 ± 13.07
Stance phase (left leg) [%]	45.91 ± 9.6	49.32 ± 12.01	51.31 ± 13.09

### 3.3 Human joint kinematics during overground walking

Likewise, the peak joint angles during unassisted walking and exoskeleton-assisted gait are presented in Table 2. Significant differences from the unconstrained condition were found for the peak knee flexion on the right leg (i.e., the actuated side) during both operation modes: TM (*ρ* = 0.001) and SC (*ρ* = 0.000). Also, the left side, i.e., the inactive side, shows a significant reduction in peak knee flexion during TM (*ρ* = 0.01). On the other hand, hip kinematics remains untouched while being assisted by the AGoRA exoskeleton since no noteworthy differences were found while the peak hip flexion/extension values lie close to typical values found for healthy subjects during unconstrained overground walking (Ward et al., 2017; Fukuchi et al., 2018).

**TABLE 2:** Lower-limb kinematics calculated by the 3D-motion analysis system Vicon for all experimental conditions assessed: Unassisted, Transparent Mode (TM), and Stance Control (SC) (mean ± std). Asterisks indicate significant differences from unassisted condition (Bonferroni, *p* < 0.05).

Parameter	Unassisted	TM	SC
Peak knee flexion (right leg) [°]	−66.80 ± 5.59	−47.33 ± 7.10*	−46.48 ± 5.44*
Peak knee flexion (left leg) [°]	−64.02 ± 5.65	−52.37 ± 9.46*	−53.84 ± 11.47
Peak hip flexion (right leg) [°]	26.00 ± 4.62	25.62 ± 7.35	21.97 ± 9.45
Peak hip flexion (left leg) [°]	24.74 ± 4.67	25.04 ± 4.94	26.30 ± 7.71
Peak hip extension (right leg) [°]	−17.22 ± 4.41	−11.58 ± 6.08	−13.86 ± 7.28
Peak hip extension (left leg) [°]	−17.38 ± 4.02	−17.00 ± 3.58	−18.83 ± 5.22

Additionally, the user survey regarding their satisfaction towards the AGoRA exoskeleton controlled by the proposed approaches produced the following outcomes: weight: 4 ± 1, dimensions: 3 ± 1, adjustment (meaning the user’s perception towards the system used to attach the exoskeleton to their limbs, i.e., the fastening system and telescopic bars): 3 ± 1, safety: 5 ± 0.25, stability (meaning how close the subject felt as though they were about to fall): 4 ± 0.25, durability: 3 ± 1.25, ease of use (i.e., how intuitive it is to use): 4 ± 1, and comfort: 3 ± 0.25, within a range between 0 and 5.

Based on the experiences gained during the experimental trials, further use with the designed platform would require a therapist or assistant well aware and capable of adjusting and mounting the exoskeleton on the user. The total time needed for the donning process was found to be around 25–30 min when the orthosis is being used for the first time since more time is required to measure the subject’s anthropometric measurements and properly adjust the length of thigh and shank segments.

## 4 Discussion

This work presents the performance assessment of the AGoRA exoskeleton during two experimental phases. The first phase evaluates the SC response with a healthy user performing a 6MWT over a treadmill in terms of angular position, angular velocity, detected gait phase, and controller gain. The second phase features the short-term effects on the walking pattern of six healthy subjects during overground walking. This lower-limb active orthosis operates using a TM that suppresses the dynamics of a non-backdrivable structure to some extent, and an SC that is meant to provide support to both knee and hip joints during the stance phase whereas it allows free movement during the swing phase. This SC approach varies hip and knee impedances on the basis of two anthropometric measurements: height and weight. Such consideration has been widely taken into account in previous designs of SC orthoses (Rafiaei et al., 2016; Villa-Parra et al., 2017). However, by using an adaptive method based on a machine-learning model and a minimal number of inertial sensors for gait phase estimation purposes, we expect to further research the effects of this control strategy on healthy subjects.

First, the treadmill-walking experimental phase served as a pilot study to evaluate the overall performance of the device and to fix minor hard- and software bugs before conducting experimental trials with it operating in a more autonomous manner (i.e., overground walking). The angular velocity profiles exhibited for both assisted joints are the result of stiffness values generated through the SC gain (varied as a function of the detected gait phase, as explained in Section 2.3.3) and show a pattern already observed in healthy subjects in previous studies (Knaepen et al., 2014). For instance, the knee modulation response (shown in Figure 10Aiv) exhibits low stiffness values during knee extension and progressively increased stiffness values during knee flexion, as reported in (Shamaei et al., 2013b). Likewise, the modulation method response for the hip joint (shown in Figure 10Biv) is in agreement with previous work as high stiffness values are applied during the HS gait phase (Huang and Wang, 2016; Akl et al., 2020).

And so, during the overground-walking experimental phase, regarding spatiotemporal gait parameters, only the gait velocity appeared to be significantly affected by the exoskeleton while operating in SC. Even though a slight reduction in gait velocity during robot-assisted training is not ideal, previous studies have demonstrated that this parameter can reduce up to 0.57 m/s while wearing an orthosis commanded by an SC approach (Rafiaei et al., 2016). Also, the gait velocity obtained applying SC is close to further results registered in (Knaepen et al., 2014; Villa-Parra et al., 2017; Ortlieb et al., 2019). Thus, such a variation seems not to be critical, also because further gait parameters did not demonstrate any significant influence by the exoskeleton while they remained close to those which are considered typical among able-bodied individuals. For instance, a study with similar experimental conditions to those imposed in this study found cadence to be around 79.7 ± 3.66 steps/min, whereas the step duration was equal to 0.76 ± 0.04 and 0.75 ± 0.03 s for right and left legs respectively, during normal overground walking (Knaepen et al., 2014). Furthermore, a study of gait analysis using an active knee orthosis reports the swing phase to be between 36% and 51% of the gait cycle (Arazpour et al., 2016), thus complying with the stance phase percentages found in this work for exoskeleton-aided walking (as may be seen in Table 1). Finally, although the stride length decreases between unassisted and assisted conditions, it remains in range with the parameters estimated for the AUTONOMYO exoskeleton (Ortlieb et al., 2019), for instance.

Regarding lower-limb kinematics, at least one significant difference per side was found for the case of the peak knee flexion, with a mean peak value of approximately 46,48° during the SC condition. Normally, the human knee joint needs 67° of flexion during the swing phase in healthy subjects during normal walking. However, the maximum knee flexion is highly dependent on the user’s gait speed. Knee peak values close to 67° have been registered when users walk at approximately 1.25 *m*/*s*, whereas values between 40° and 50° are associated to gait velocities near 0.5 *m*/*s* (Asseldonk et al., 2008). Even more, using an SC orthosis has proven to reduce this value to 40° in healthy subjects (Arazpour et al., 2014). Such outcomes along with other studies, where the knee stiffness is controlled through robot-aided assistance and similar knee kinematics are displayed (Shamaei et al., 2013a; Cestari et al., 2015; Ortlieb et al., 2019), suggest that the influence of the AGoRA exoskeleton on the user’s knee is in accordance with what is found in the literature. In the same way, the peak hip flexion and extension registered during the SC assessment are similar to those presented in (Asseldonk et al., 2008), HUALEX exoskeleton (Tran et al., 2016), and the unilateral orthosis reported in (Hussain et al., 2013).

For this particular setting, such a reduction in terms of knee angular displacement seems to be attributable to some hardware issue (in particular, to the fastening system), since the left side, which has no actuation mechanism assembled and thus perceives no electromechanical activation, also shows a significant reduction in peak knee flexion during TM. An improper attachment to the user’s limbs mainly produces joint misalignments which are a well-known problem when dealing with physically coupled systems, e.g., humans wearing exoskeletons (Gordon et al., 2018). If perfectly-aligned joints are assumed, exoskeleton forces can be modeled as equal. Nevertheless, the presence of joint misalignment results in the imperfect transmission of torque from the exoskeleton to the user’s body (Schiele and van der Helm, 2006), thus introducing undesirable forces parallel to the human limb which can cause discomfort or unintended changes in the behavior of the control system. In spite of this, hip kinematics present no considerable change from the baseline condition, which may imply a better attachment to the pelvic region, and thus, better compliance of the exoskeleton’s control system on the hip joint. Overall, it cannot be ruled out that the differences among experimental conditions could have been much lower if the users had been fitted with an optimal orthosis system.

Concerning user satisfaction, results show that the lowest score (3) was related to the items regarding “dimensions”, “durability”, “comfort”, and “adjustment”. It is worth noting that the insight concerning dimensions might be related to the inherent protrusion of the actuation mechanisms which are placed laterally, and whose bulkiness is even more notorious in a unilateral device such as the version proposed for this study. Likewise, the comfort factor in wearable devices has been commonly associated with features such as sensors, straps, and weight (Huo et al., 2016). Besides, offering unilateral hip and knee assistance for healthy subjects may be an additional factor that promotes the discomfort experienced by the participants, since some of them expressed feeling some pain in the upper back after wearing the exoskeleton. Physiological theories have been developed to address these limitations (Li et al., 2015), but this issue remains a major problem for powered autonomous orthoses. Finally, the remark on the adjustment system seems to be in accordance with the analysis drawn from the kinematic outcomes and thus encourages some hardware modifications to obtain a more robust system, e.g., new materials able to properly adjust the exoskeleton and to suppress relative movement between human and machine.

Finally, it is important to note that this study is limited by the reduced number of subjects recruited for the study, which compromises to some extent the power of the applied statistical tests. However, since the participants were rather homogeneous in terms of age and anthropometric measures due to the reduced operating spectrum that the exoskeleton provides, the outcomes should allow the debugging of several hard and software issues (discovered during experimental trials) for further iterations of the device and future trials with mobility-impaired patients. Besides, although the results found in terms of spatiotemporal gait parameters and lower-limb kinematics are similar to those presented in the literature, the use of a single sensor unit attached to the user might be useful for clinical settings where wearability is critical and donning times are expected to be reduced.

## 5 Conclusion

The performance evaluation of the AGoRA exoskeleton in the short term in healthy subjects has been featured in the present study. Six neurologically-intact subjects were recruited to perform several overground trials under three experimental conditions: unassisted walking, and exoskeleton-assisted walking while operating in TM and SC. Spatiotemporal gait parameters and lower-limb kinematics were processed from measurements captured by a motion capture system based on passive optical markers. Additionally, in order to assess the exoskeleton’s performance both objectively and subjectively, a user survey was conducted to collect data regarding their satisfaction with the implemented technology.

Most spatiotemporal parameters did not exhibit any significant change from the unassisted condition for both operation modes, and only knee kinematics was compromised while the user was wearing the exoskeleton. However, given the fact that the sample size of the present study does not guarantee statistical power, the results here presented should be taken with caution and should not be considered as definitive proof of the efficiency of the approach implemented. Furthermore, an improper attachment to the subject’s limbs, as a consequence of a deficient fastening system, did not ensure kinematic compatibility and could have influenced the effect of the torque profiles coming out of the control system.

Thus, the research conducted in this study together with the results obtained should only serve as a preliminary evaluation to validate the alterations in the gait pattern generated by the non-backdrivable exoskeleton applying a control strategy based on stance control. The gait alterations for an entire significant population are not discussed in this study. On the other hand, this work is not intended to prove its effectiveness within a gait rehabilitation program. Although the gait pattern of healthy subjects seems to remain unaffected by the actuation of the exoskeleton, further studies should involve actual stroke survivors so that a real comparison with respect to traditional therapy is feasible. To this end, a larger sample size is recommended. It has been estimated that at least 384 participants should be involved in the future to achieve a confidence level of 95% and an error margin of ±5%. Also, further modifications of the device should be carried out in the hardware and software architectures to facilitate its donning and command by healthcare professionals within a clinical setting, such as the improvement of the fastening system to make it easier to adjust and more robust to misalignments, and the development of a graphical user interface.

## Data Availability

The original contributions presented in the study are included in the article/Supplementary Material, further inquiries can be directed to the corresponding author.
